# Genomic patterns of strain-specific genetic structure, linkage, and selection across fall armyworm populations

**DOI:** 10.1186/s12864-025-11214-8

**Published:** 2025-02-07

**Authors:** Ashley E. Tessnow, Rodney N. Nagoshi, Robert L. Meagher, Todd M. Gilligan, Ben M. Sadd, Yves Carrière, Holly N. Davis, Shelby J. Fleischer, Kelly Richers, John C. Palumbo, Patrick Porter, Jose Carlos Verle Rodrigues, Gregory A. Sword

**Affiliations:** 1 Department of Entomology, Texas A&M University, College Station, TX United States of America; 2 Center for Medical, Agricultural and Veterinary Entomology, Department of Agriculture-Agricultural Research Service, Gainesville, FL United States of America; 3 Department of Agricultural Biology, Colorado State University, Fort Collins, CO United States of America; 4 School of Biological Sciences, Illinois State University, Normal, IL United States of America; 5 Department of Entomology, University of Arizona, Tucson, AZ United States of America; 6 Texas A&M AgriLife Research and Extension, Weslaco, TX United States of America; 7 Department of Entomology, The Pennsylvania State University, University Park, PA United States of America; 8 The Wedge Entomological Research Foundation, Bakersfield, CA United States of America; 9 Department of Entomology, University of Arizona, Yuma, Arizona, United States of America; 10 Texas A&M AgriLife Research and Extension, Lubbock, TX United States of America; 11 Insect Management and Molecular Diagnostics Lab, Department of Agriculture - Animal and Plant Health Inspection Service, Edinburg Texas, United States of America

**Keywords:** *Spodoptera frugiperda*, Population genomics, Strain divergence, Z-chromosome

## Abstract

**Background:**

Molecular genetic approaches have become vital to understanding the evolutionary processes that act on insect pest populations. From mapping the development of resistance to monitoring and predicting pest movement, genomic tools can inform and enhance pest management programs. Here, we used whole genome sequencing population genomics to unravel novel patterns of population structure, linkage, and selection across the genome of a notorious agricultural pest, the fall armyworm.

**Results:**

Our data strongly support the existence of two genetically distinct strains of fall armyworm in North America, which have previously been referred to as the C-strain and the R-strain. Although these strains have diverged genetically, we find that differentiation is not uniform across the genome. The Z-chromosome appears to drive divergence between strains with high levels of linkage observed across this chromosome. We also show that a region of the Z-chromosome containing a circadian clock gene implicated in allochronic reproductive isolation is under strain-specific selection. Our data indicates that strains differ in their geographic distributions and exhibit distinct patterns of geographic sub-structuring indicative of unique dispersal patterns. We provide the first evidence for nuclear genomic differentiation between the two major overwintering populations of fall armyworm in the US. Finally, our data reveal population-specific patterns of selection on genomic regions containing putative insecticide resistance alleles, which could relate to their biogeography.

**Conclusions:**

Our results support the existence of the fall armyworm as a pest dyad in the US, with genetically-distinct strains differing in their population structure, dispersal patterns, and genomic signatures of selection on regions likely involved reproductive isolation and insecticide resistance. These differences should be considered when devising and implementing management strategies.

**Supplementary Information:**

The online version contains supplementary material available at 10.1186/s12864-025-11214-8.

## Background

Understanding patterns of population structure, movement, and selection in agricultural insect pests can be key to developing effective control measures that reduce economic and environmental costs. Molecular tools and genomic analyses have become essential for understanding, predicting, and combatting insect pest problems. For example, molecular markers, including those derived from whole genome sequencing (WGS), were used to monitor the development and spread of resistance to commonly used insect control strategies [1–3] and identify the origin of novel pest infestations [4–6]. With ongoing advancement in sequencing technologies and cost reductions, it is now feasible to implement large-scale WGS-based population genomic studies to unveil elusive patterns of population structure, movement, and selection in various insect pests.

The fall armyworm, *Spodoptera frugiperda* (J.E. Smith), is a noctuid moth native to the Western Hemisphere that is invasive throughout sub-Saharan Africa, Asia, Australia, and New Zealand [7]. This species has a host range of over 353 different plant species and is an agricultural pest of global concern [8]. Despite a diverse host range, fall armyworms show a preference for gramineous hosts such as corn, sorghum, pasture grasses, cereals, and turf, where they can cause significant economic damage.

In the Western Hemisphere, the fall armyworm comprises two morphologically identical but genetically and behaviorally distinct strains (C- and R-) [9–12]. Slight differences in host ranges have been reported between strains; in South America, the C-strain is primarily associated with corn and sorghum, and the R-strain is primarily associated with pasture and turf; in North America, the R-strain exhibits a broader host range that includes corn and sorghum [13]. The two strains also exhibit different activity patterns in the adult stage, with C-strain moths mating early in the night and R-strain moths mating closer to dawn [12, 14, 15]. Hybridization between strains occurs in laboratory studies [16–20]. However, field studies based on mitochondrial and nuclear strain markers indicate unidirectional mating incompatibility between strains that limits post-hybridization gene flow. In these cases, hybrid females with maternally inherited R-strain mtDNA successfully mate with C-strain males and backcross into the C-strain population. However, hybrids with C-strain mtDNA rarely if ever backcross successfully to the R-strain [12, 21–23]. In addition to these behavioral differences and barriers to gene flow, the strains also exhibit differential susceptibility to commonly used control measures such as pyrethroids and *Bacillus thuringiensis* (Bt) crops [24–26], although these studies have not yet expanded to include more recent insecticide chemistries.

Unlike many noctuid moth species, neither fall armyworm strain can overwinter in diapause. Therefore, this species only survives the winter in locations warm enough to support continuous generations. In the United States, overwintering has only been documented in the southern tips of Texas and Florida. As temperatures rise during the spring, fall armyworm undergoes a stepwise northward range expansion, ultimately extending its range into Canada by the end of the growing season. Geographic population structure and movement patterns of fall armyworms from their overwintering sites have been exclusively studied in the C-strain using mitochondrial haplotypes [27–30]. Using these haplotypes, it was found that fall armyworm overwintering in Florida infests regions east of the Appalachian Mountain range (i.e. the eastern flyway), whereas fall armyworm overwintering in Texas migrates across most of the continent between the Rocky and Appalachian Mountains, as well as into locations north of the Appalachians including the mid-Atlantic region, New England, and Canada (i.e. the central flyway) [31]. Although fall armyworms are routinely captured in the western United States including Arizona and California (i.e., the western flyway), only a single study has assessed movement into this region with a focus on Arizona [32]. Additionally, while it is often assumed that R-strain fall armyworms follow the same annual migration trajectory across the continental US as their C-strain counterparts, this has never been assessed formally.

Because the fall armyworm as a species is a major pest, several control strategies have been deployed to manage populations across its native range. These strategies include planting Bt transgenic crops, and spraying pyrethroid, organophosphate, and diamide chemical insecticides. Field-evolved resistance to these control strategies has been a concern and is often associated with an increase in detoxification or metabolic resistance [33–35]. This includes changes in the expression of key detoxification enzymes such as cytochrome p450s (CYP) and glutathione S transferases (GST) [35, 36]. Resistance to diamides has also been reported through target site mutations in the ryanodine receptor (RYR) [37, 38].

In this study, we used a whole genome sequencing approach to comprehensively assess the genetic structure and movement patterns of the fall armyworm in the United States (US). We first assessed the population structure of fall armyworms across the US, specifically considering the interactive effects of strain and geography on population differentiation. This included a novel assessment of fall armyworm populations in the western US. We then evaluated the distinct genomic patterns of linkage and differential selection that occur across unique fall armyworm populations. We consider the implications of our results for fall armyworm migration and management.

## Methods

### Sample collections

Male fall armyworm moths were collected using universal bucket traps (Unitraps) baited with either a 2-component (Scentry Biologicals, Inc., Billings, MT, USA) or 3-component (Trécé, Inc., Adair, OK, USA) fall armyworm pheromone lure [39]. All sampling locations, dates, and predominant nearby host plants are listed in Table 1. Fall armyworm strains do not significantly differ in their attraction to either pheromone lure, so pheromone selection is not expected to be a source of bias in our collection data [40]. For downstream analyses, moths collected from Florida and Georgia were defined as the eastern flyway, moths collected from Texas, Illinois, Colorado, and central Pennsylvania were defined as the central flyway, and moths collected in Arizona and California were defined as the western flyway [31]. After collection, moths were preserved in > 70% ethanol, shipped to Illinois State University, and stored at -20 °C until DNA extraction.

**Table 1:** Collection information for fall armyworm samples analyzed as part of this study. All individuals were collected in 2021 in pheromone baited moth traps. When host plants are designated as mixed agriculture, trap collections occurred in field sites where multiple crops including but not limited to maize, sorghum, sugarcane, peanut, soybeans, alfalfa, sunflower, hemp, vegetables and small grains were planted in close proximity

County	State	Flyway	Month	Host Plants	*n*	# C-strain	# Hybrid	# R-strain
Hidalgo	TX	Central	March	Sorghum	16	5	0	11
Hidalgo	TX	Central	June	Sorghum	17	10	0	7
Hidalgo	TX	Central	October	Maize	17	3	0	14
Lubbock	TX	Central	October	Mixed agriculture	21	13	0	8
Tazewell	IL	Central	August	Maize	16	0	0	16
Tazewell	IL	Central	September	Mixed grasses	5	0	0	5
Larimer	CO	Central	September	Mixed agriculture	19	17	0	2
Centre	PA	Central	August	Mixed agriculture	17	3	0	14
Miami-Dade	FL	Eastern	March	Maize	12	4	0	8
Palm Beach	FL	Eastern	March	Mixed agriculture	27	9	3	15
Palm Beach	FL	Eastern	September	Mixed agriculture	17	7	0	10
Palm Beach	FL	Eastern	October	Mixed agriculture	22	4	2	16
Palm Beach	FL	Eastern	November	Mixed agriculture	19	3	0	16
Duval	FL	Eastern	October	Mixed grasses	15	3	0	12
Burke	GA	Eastern	September	Mixed agriculture	19	1	0	18
Burke	GA	Eastern	October	Mixed agriculture	18	17	0	1
Pike	GA	Eastern	July	Maize	12	0	2	10
Juana Diaz	PR	Eastern	March	Maize	21	20	1	0
Pinal	AZ	Western	September	Alfalfa	10	10	0	0
Pinal	AZ	Western	September	Maize	10	10	0	0
Yuma	AZ	Western	September	Mixed agriculture	21	21	0	0
Kern	CA	Western	June	Mixed grasses	16	15	1	0
Kern	CA	Western	August	Mixed grasses	23	23	0	0
Kern	CA	Western	September	Mixed grasses	22	22	0	0

### DNA extraction

DNA was extracted from the thorax of each moth sample. After excision, thorax tissues were snap frozen in liquid nitrogen, and then macerated using a sterilized plastic pestle. DNA was extracted using the Gentra Puregene Tissue Kit (Qiagen) in strict accordance with the manufacturer’s protocol. Following extraction, DNA was purified by adding 7.5 µl of 3 M sodium acetate and 180 µl of chilled 100% ethanol to each sample. Samples were incubated overnight at -20 °C and then centrifuged for 25 min at 4 °C. The supernatant was removed, and the pellet was washed with 500 µl of chilled 75% ethanol. Samples were centrifuged for 5 min at 4 °C and then air dried for 25 min. 100 µl of molecular grade H_2_O was used to elute the pure DNA. After DNA was resuspended, samples were stored at -20 °C before being sent to the Texas A&M AgriLife Genomics and Bioinformatic Service (TxGEN) for library preparation and sequencing.

### DNA sequencing

Sample libraries were prepared using the NEXTFLEX Rapid XP kit (PerkinElmer) which was automated on a Sciclone NGSx liquid handler (PerkinElmer). Library preparation was followed by SPRI bead cleanup and size selection. All fragments between 520 and 720 bp were amplified for 10 PCR cycles. After amplification, another round of SPRI size selection was carried out to retain DNA fragments larger than 450 bp. Libraries were diluted to a final concentration of 2.25 ng/µl using the JANUS liquid handler (PerkinElmer). Libraries were sequenced using a 2 × 150 bp run in a single lane of an Illumina NovaSeq S4 XP flowcell (Illumina). After sequencing, the raw reads were demultiplexed, and adapters were removed using bcl2fastq 2.20 (Illumina).

### Strain determination

The strain of each sample was determined by identifying known strain-specific markers, including *Tpi* [41, 42] and three diagnostic SNPs [43] from raw sequencing reads. Individuals with fewer than 2 diagnostic markers sequenced were removed from the analysis to ensure we had confidence in our strain calls. In total, 15 individuals (3.5%) were removed due to lack of conclusive strain information, with no more than 3 individuals removed from any single collection location. If individuals were heterozygous for 1 or more of the diagnostic markers, then the individual was called as a putative hybrid. In total, we were able to determine the strain of 412 moth samples (Table 1).

### Variant calling, imputation, and filtering

The initial genome mapping and variant calling were conducted by TxGEN using the Dragen-GATK V 2.0 pipeline and default parameters. This involved using a Smith–Waterman algorithm to map raw reads to the fall armyworm chromosome-level genome assembly published by Gimenez et al. (2020) [44]. Reads were then sorted and duplicates were marked using a picard algorithm. Variant calling was conducted using GATK. BCFTools v 1.14 [45, 46] was then used to filter out variants with sequencing depth less than 6, a minimum genotype quality score less than 10, and more than 50% missing data. After filtering, missing genotypes were imputed with Beagle v 4.1 [47]. All imputed genotypes with a genotype probability score less than 0.9 were removed to increase our confidence in the imputed markers.

After imputation, the .vcf file was uploaded to the Texas A&M High Performance Research and Computing GRACE cluster for analysis. All scaffold positions were renamed to their NCBI chromosome numbers (chromosomes 1–30 were the autosomes and the Z-chromosome was named chromosome 31). VCFtools v 0.1.16 [48] was used to remove all loci that did not map to one of the 31 chromosomes. After filtering, our full dataset contained 3,318,420 SNPs. This full dataset was used in selective sweep analyses. We then used VCFtools to filter SNPs with more than two alleles and that had a global minor allele frequency (maf) of < 0.01. This reduced biallelic dataset contained a total of 1,924,998 SNPs and was used for all other analyses of population structure.

### Identifying genetic structure and assessing strain divergence

Individuals identified as interstrain hybrids were removed from the dataset, and Weir and Cockerham weighted F_st_ values between strains were calculated at each individual SNP marker and within 10 kb sliding windows across the genome using VCFtools. A Manhattan plot visualizing genomic F_st_ values for each individual SNP was created in R/ggplot2. Additional line plot visualizations showing F_st_ values across 10 kb sliding windows within each chromosome were created in R/ggplot2. Because there was a large concentration of F_st_ values nearing 1 on the Z-chromosome, we used the program Asaph [49] to test for a structural rearrangement in the dataset.

Previous work has shown that loci on the Z-chromosome likely play a disproportionate role in strain divergence relative to autosomal loci [43]. Therefore, we assessed population structure separately for the autosomes and the Z chromosome. For this analysis, three .vcf files were converted to plink binary file formats using Plink v. 1.9 [50], and then to eigenstrat format using the convertf tool within EIGENSOFT v. 7.2.1 [51]. The smartpca tool in EIGENSOFT was used to run a smart principal component analysis on each of the three variant datasets (all data, autosomes, and Z-chromosome). Pairwise F_st_ values for each population defined a priori as either strain, flyway, or strain x flyway were calculated as part of the smartPCA run.

Because high divergence was noted on the Z-chromosome, we calculated absolute sequence divergence (Dxy) across this chromosome to identify regions that are genetically divergent between strains, also known as genomic islands. To do this, our initial sequencing data was aligned to the Z-chromosome using bwa. We then used SAMTOOLS mpileup [46] to create a new VCF file that contained information about every locus on the Z-chromosome (variant + invariant sites). This dataset was filtered using VCFTools to remove nine putative hybrid individuals and all sites with more than 20% missing data. Dxy values were calculated in 10 kb sliding windows using Pixy v 1.2.5.beta1 [52]. A kernel-based smoothing algorithm was applied to the resulting windowed Dxy values. The order of Dxy values was permuted 10,000 times and the same kernel-based smoothing algorithm was applied to the permuted data. Significantly elevated Dxy values, indicative of genomic islands, were identified as regions of the chromosome where our smoothed line exceeded the most extreme value from our permutation distribution [53, 54].

### Calculating nucleotide diversity, tajima’s D, and linkage disequilibrium

The biallelic .vcf file was split into a file containing only the autosomes (1,752,217 SNPs) or only the Z-chromosome (172,781 SNPs). These files were further divided by strain for a total of seven datasets: [1] all autosomal and Z-chromosome SNPs [2], all autosomal SNPs [3], all Z-chromosome SNPs [4], C-strain autosomal SNPs [5], C-strain Z-chromosome SNPs [6], R-strain autosomal SNPs, and [7] R-strain Z-chromosome SNPs. Tajima’s D, and Nucleotide diversity (π) was calculated in 5 kb sliding windows for all seven datasets. Windows of 5 kb contain an average of 24 SNPs per window. Linkage disequilibrium (LD) was also calculated for all datasets for SNPs located at eleven different distance intervals: 100 bp, 250 bp, 500 bp, 1,000 bp, 2,500 bp, 5,000 bp, 7,500 bp, 10,000 bp, 25,000 bp, 50,000 bp, and 75,000 bp. To increase the number of SNPs assessed at each interval, all distance intervals included a range of plus or minus 50 bp. LD was then averaged within each interval and plotted against distance for each dataset. All .vcf file filtering, Tajima’s D, nucleotide diversity, and LD calculations were done using VCFtools v 0.1.16. All plots were created using R/ggplot2.

### Identifying selective sweeps

The full dataset containing 3,318,420 SNPs was first split into two files containing either C-strain or R-strain individuals. Because our smartpca indicated geographic sub-structuring within the C-strain, the C-strain .vcf file was further split by sub-population creating four unique datasets to run selective sweep analyses; (1) all R-strain individuals, (2) C-strain individuals from Texas (i.e. the central flyway), (3) C-strain individuals from Florida and Georgia (i.e. the eastern flyway), and (4) C-strain individuals from Puerto Rico. Finally, each dataset was split by chromosome, creating 31 files for each genetically distinct population. All .vcf file splits were done using VCFtools v 0.1.16.

Hard selective sweeps were first identified using the program SweeD which utilizes a site frequency spectrum (SFS) method for detection [55]. SweeD computes a composite likelihood ratio (CLR) to identify regions within the chromosome that have decreased genetic diversity indicative of genetic hitchhiking. CLR ratios were calculated for 5 kb regions. This sliding window was set for each chromosome by dividing the chromosome length by 5 kb and then setting this value as the grid size. Manhattan plots visualizing CLR values across each genomic window were created in R/ggplot2. Outlier sites were defined as any chromosomal window within the 99.99th percentile of CLR values. For each chromosome with outlier sites detected, SweeD was rerun using 1 kb sliding windows. The CLR value was evaluated for windows surrounding the outlier site, and the sweep region was identified as the first and last consecutive window with elevated CLR (in the top 99th percentile of CLR values). Annotated genes within this selected region were then identified from the OGS6.1 transcriptome (https://bipaa.genouest.org/sp/spodoptera_frugiperda_pub/download/annotation/corn/OGS6.1/) using IGV 2.16.0.

After identifying selective sweeps using the site frequency spectrum method (i.e. SweeD), we utilized the program OmegaPlus which is a linkage-based method for detecting selective sweeps [56]. This method uses the ω statistic to identify patterns of excess LD in windows across the genome. SFS and LD based methods are complimentary, rarely identifying the same regions under selection. Utilizing both methods can increase the detection of genomic regions under selection. OmegaPlus was run using the same .vcf files that were used with SweeD. However, because LD was found to be higher on the Z-chromosome compared to the autosomes, an LD-method for selective sweep detection could result in many false positives. Therefore, this analysis was only preformed using the autosomes. This program was once again run in 5 kb sliding windows with the minimum window size set to 500, the maximum window size set to 2,500 and the LS method set to Rsquare. Because LD-based methods such as OmegaPlus can have a higher false positive rate for identifying selective sweeps, outliers were identified by visual inspection. There were no cases in which consecutive windows had elevated omega value, therefore genes were only annotated within the 5 kb outlier window. Manhattan plots visualizing ω across each genomic window were created in R/ggplot2.

## Results

### Strain distribution

**Fig. 1:**
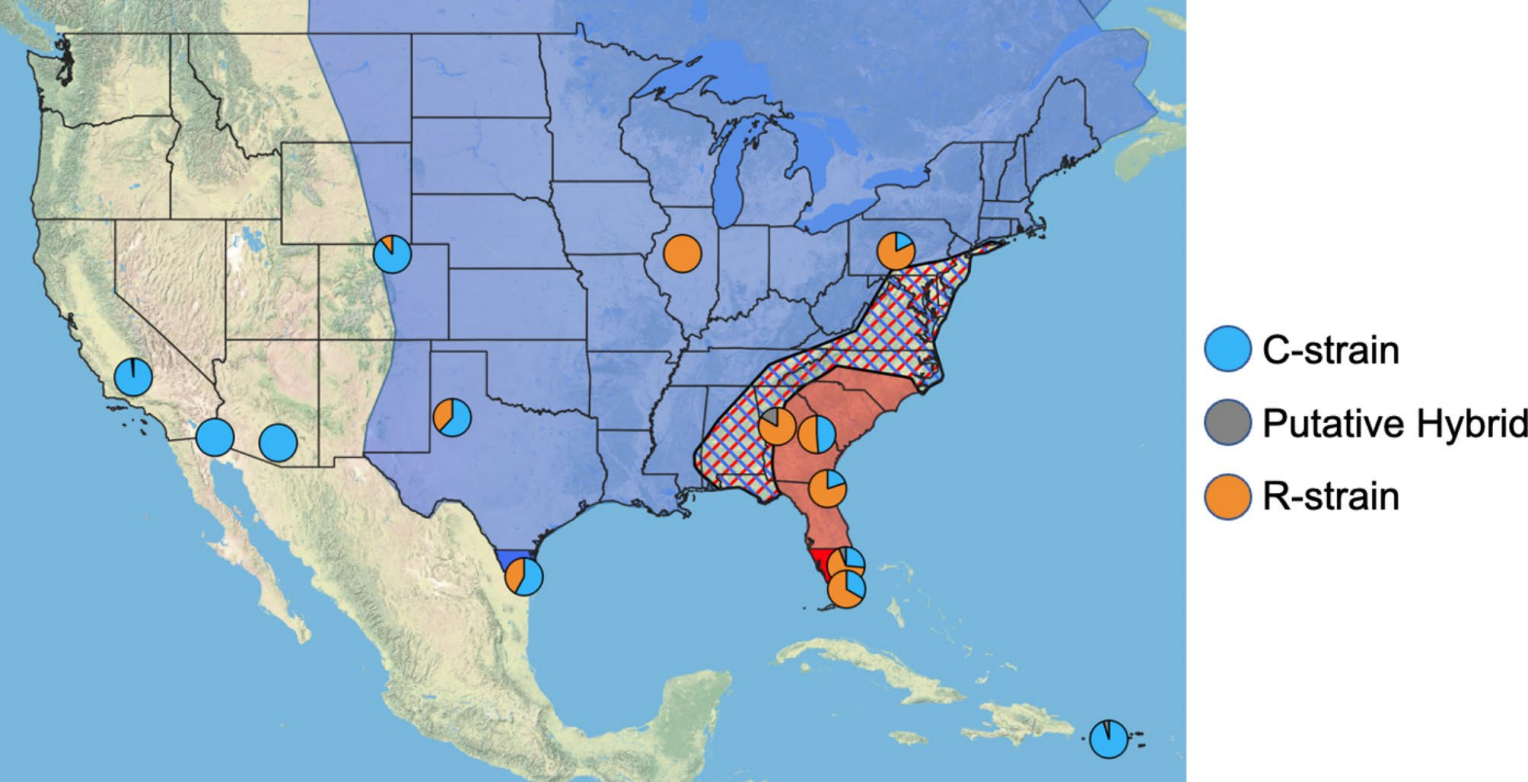
The relative proportion of C-strain, R-strain, and putative hybrid individuals collected at each location. When locations were sampled at multiple timepoints, we did not observe any notable differences in strain composition across collections, so all individuals throughout the year are pooled within each location. Background shading designates western (clear), central (blue), and eastern (red) flyways. Hashed background designated area of intermixing between the central and eastern flyways

The relative proportion of fall armyworm strains differed across geographic regions in our study (Fig. 1). Both strains were abundant in the central and eastern flyways, whilst the C-strain dominated Puerto Rico and the western flyway. In the eastern flyway, we sequenced 159 samples collected across multiple locations and time points. In total, 30% were C-strain, 66% were R-strain and 4% were putative hybrids. In the central flyway, 128 individuals were collected and sequenced across multiple locations and timepoints. This flyway was composed of 48% C-strain, 52% R-strain, and 0% putative hybrid individuals. We only sequenced 21 individuals from Puerto Rico collected at a single time point and the collection comprised 95% C-strain, 0% R-strain, and 5% putative hybrids. Since this was a relatively limited collection, this may not represent accurate strain demographics across the island. In the western flyway, we sequenced 102 individuals sampled across three locations in multiple seasons. These moths were collected near a variety of host plants including maize, mixed grasses, and alfalfa. Nevertheless, we found that over 99% of the population was C-strain and only one putative hybrid individual was detected. This sampling was more extensive than that of Puerto Rico, and is therefore more likely representative of the true strain composition of fall armyworms in western US in 2021.

### Genetic structure and strain divergence

**Fig. 2:**
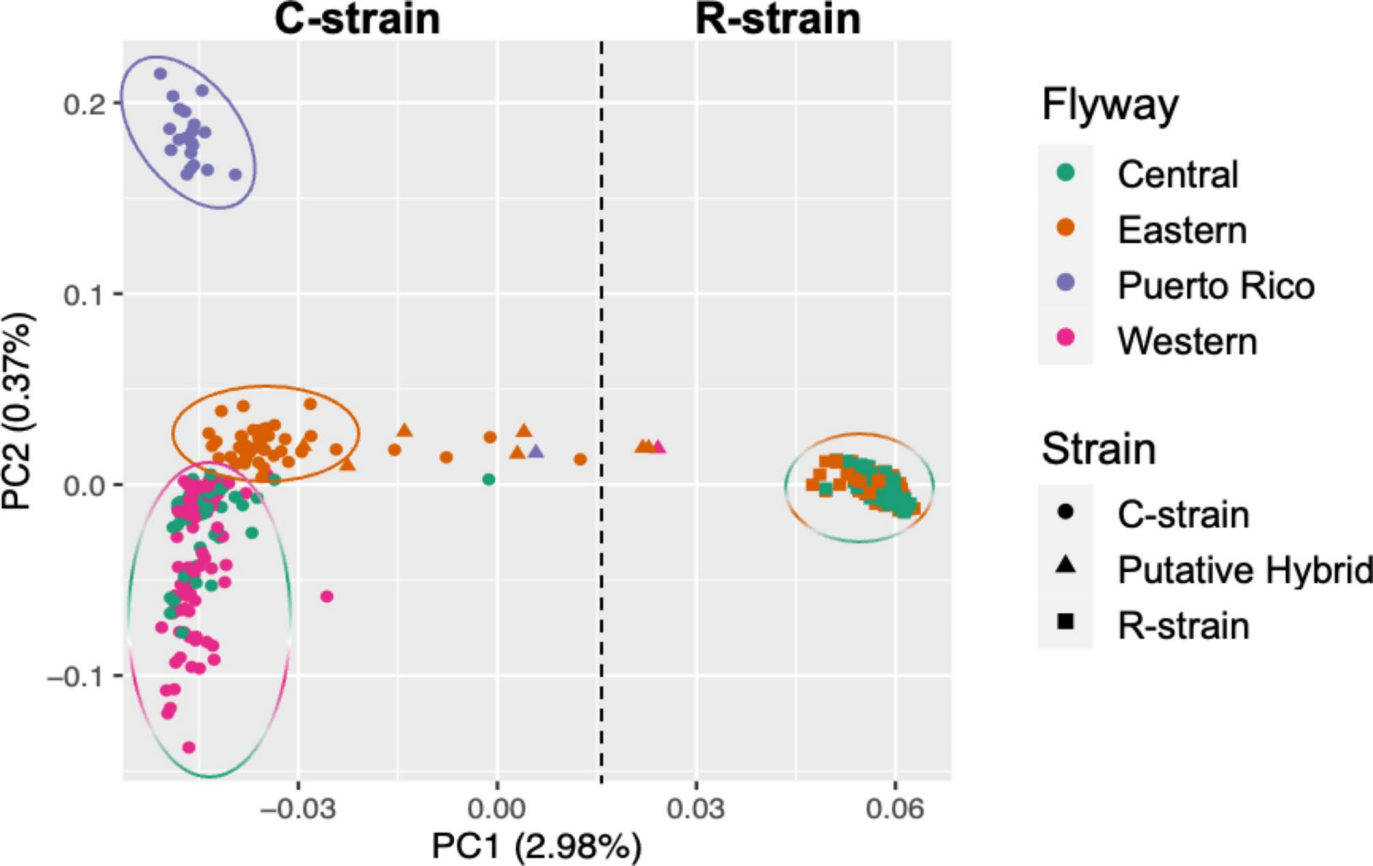
PCA visualizing patterns of autosomal genetic structure within fall armyworm samples collected in the US. Circle markers represent the C-strain, square markers represent the R-strain, and triangle markers indicate putative hybrids. Colors indicate geographic regions where samples were collected. PC1 neatly divides the C-strain from the R-strain, while geographic regions within the C-strain cluster along PC2. No geographic clustering is seen in the R-strain. F_st_ values differentiating population clusters can be found in Table S1

SmartPCA analyses were preformed to identify patterns of population structure across the US and Puerto Rico. We conducted three separate analyses using either Z-chromosome markers only, autosomal markers only, or the full dataset. While similar patterns of structure were evident across all three datasets, the linkage observed on the Z-chromosome could confound our results. Therefore, we focused our results on the autosomes (Fig. 2). The first principal component of our autosomal analysis, which explained 2.98% of the variation in the dataset, showed a clear separation of individuals by strain. The second principal component, which explained 0.37% of the variation in the dataset, clustered C-strain individuals by geographic location/flyway. Here, the western and central flyways grouped together but were distinct from individuals collected in the eastern flyway, which were also distinct from individuals collected in Puerto Rico. Although this accounts for a small percent of the variation in the dataset, the pattern was clear and indicates geographic structuring between C-strain fall armyworm populations. In contrast to the C-strain, no evidence of geographic structuring was found in the R-strain. Tracy-Widom statistics indicate that the first six principal components could explain significant genetic variation in the dataset (*p* < 0.01). After exploring all six principal components, we found that PC3 seemed to resolve very similar patterns of geographic structuring as PC2, whereas PCs 4–6 did not resolve any additional structure in the dataset (data not shown). These findings highlight the complex population structure of fall armyworms and the role of geographic factors in shaping this structure.

**Fig. 3:**
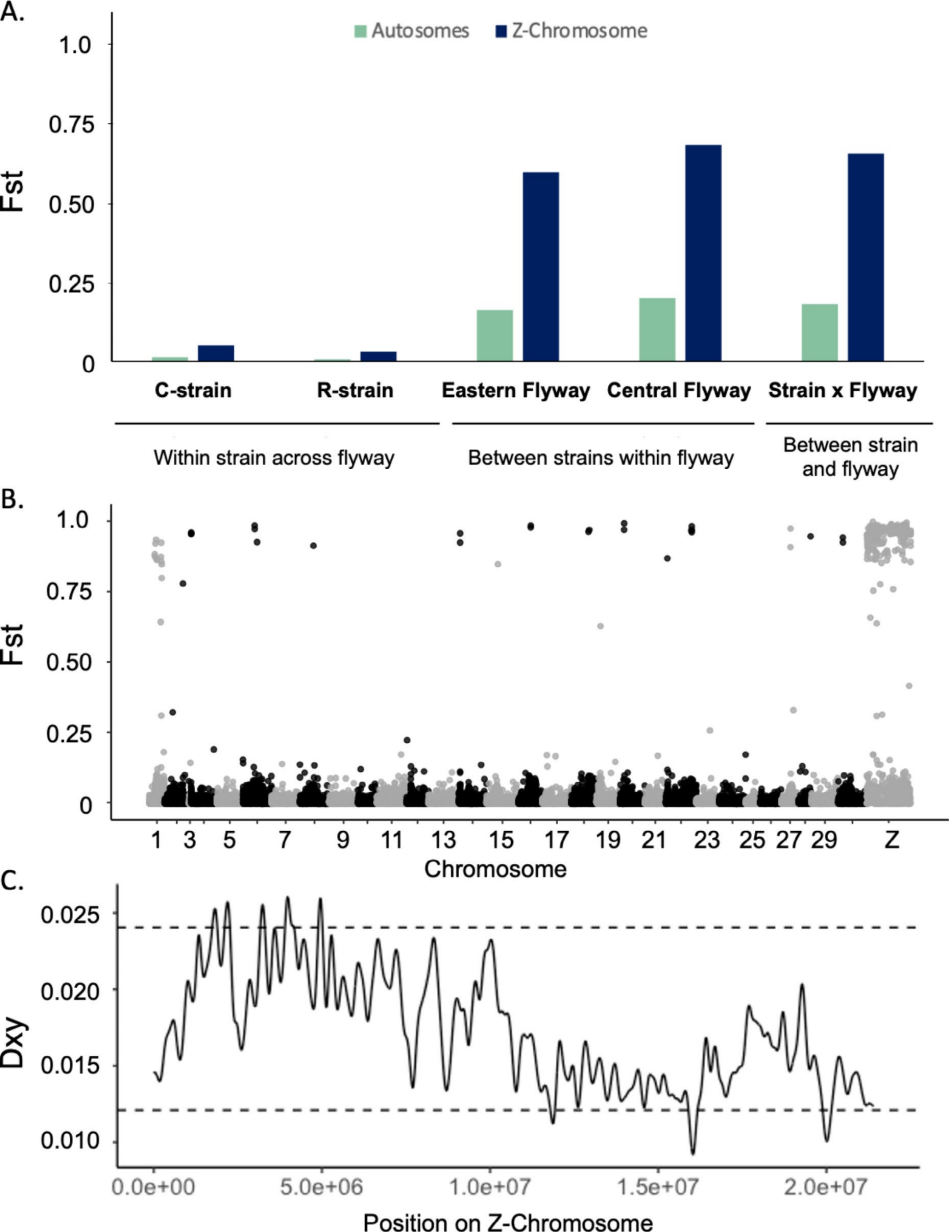
Comparison of F_st_ values between strains. **(A)** Comparison of F_st_ values calculated across the Z-chromosome and autosomes. The first comparison is within the C- and R-strains. The second comparison is between C- and R-strain individuals within a region (central or eastern flyway). The final comparison is between C-strain individuals and R-strain individuals collected in different flyways. **(B)** F_st_ values between C- and R-strain individuals calculated in 10 kb sliding windows across the genome. **(C)** Kernel smoothed absolute divergence (Dxy) between strains calculated in 10 kb windows across the Z-chromosome. Values above the dashed line exceed the maximum value of our permutation distribution and thus are significantly elevated

Weir and Cockerham weighted F_st_ values between strains differed across the genome, with much higher differentiation occurring on the Z-chromosome (F_st_= 0.671) compared to the autosomes (F_st_=0.086) (Fig. 3A & B). This was evident across 10 kb sliding windows, which showed few highly divergent regions between strains on the autosomes, but significant strain divergence across the entire Z-chromosome (Figure S1). When assessing divergence for each individual SNP, we also found that 581 of the 672 SNPs (86.5%) with F_st_ > 0.9 were located on the Z-chromosome. Despite high divergence, the program Asaph found no evidence of a large structural rearrangement between strains on this chromosome that might account for the observed difference.

When comparing the eastern and central flyways in which both strains were abundant, the genetic differentiation across the entire genome (Z-chromosome and autosomes) between strains within a flyway (F_st_=0.170) was similar to the genetic differentiation between strain across flyways (F_st_=0.189). This indicates that sympatric strains show similar levels of hybridization compared to allopatric strains (Fig. 3A). We also calculated pairwise F_st_ values between strains and flyways using SNPs located on the autosomes (Table S1) or on the Z-chromosome (Table S2). F_st_ values range from 0 to 1 and measure the extent to which genetic variation can be explained by population identity, with higher values indicating higher levels of differentiation between populations. Our data suggest that the highest levels of divergence occur between strains, with much lower levels of genetic variation being explained by regional differences within a strain. Additionally, we find that the amount of genetic variation that can be explained by strain is substantially higher on the Z-chromosome (F_st_ values ranging from 0.624 to 0.715) compared to the autosomes (F_st_ values ranging from 0.076 to 0.116).

Because both our PCA and F_st_ values indicated the Z-chromosome was playing an important role in genetic differentiation between strains, we calculated absolute sequence divergence (Dxy) across the Z-chromosome to identify genomic islands of divergence between the two strains. Dxy was significantly elevated in a region from 1,740-5,010Kb, but was elevated overall across a broader 10 kb region (Fig. 3C) that makes up nearly half of the Z-chromosome.

### Nucleotide diversity, tajima’s D, and linkage disequilibrium

**Fig. 4:**
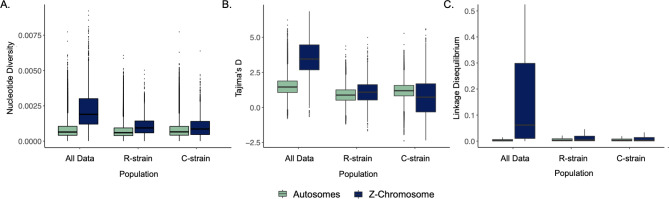
Population statistics calculated across the autosomes (green) and the Z-chromosome (blue) for all three datasets (All individuals, R-strain, and C-strain). **(A)** Nucleotide diversity (π) and **(B)** Tajima’s D were calculated in 5 kb sliding windows. **(C)** Linkage Disequilibrium (LD) was calculated for all SNP pairs separated by 10,000 bp. This is past the LD decay asymptote, but LD remains high in the combined dataset

Due to clear differences between the autosomes and Z-chromosomes, Nucleotide diversity (π), Tajima’s D (*D*) and Linkage Disequilibrium (LD) were assessed separately for these datasets. These statistics were calculated for the entire fall armyworm population that included all members of both strains, and then individually within each strain. We found that across all datasets, nucleotide diversity tended to be low, with median values ranging from 0.000596 to 0.000654 for the autosomes and 0.000858 to 0.00189 for the Z-chromosome (Fig. 4A). Although this is the first study to assess the autosomes and Z-chromosomes of the fall armyworm separately, this range is generally in line with previous studies [57, 58].

Across all datasets both the mean and median values for *D* were positive (Fig. 4B). Values of *D* were slightly higher for the combined dataset compared to the individual C-and R-strain datasets, with a median *D* of 1.46 for the autosomes and 3.45 for the Z-chromosome. While this is not surprising and indicates that balancing selection is maintaining two strains, it is noteworthy that *D* is much higher on the Z-chromosome compared to the autosomes when considering the entire fall armyworm population. In contrast, when calculated separately within the C- and R- strains, *D* was similar between the autosomes and the Z-chromosome in both strains with median values ranging from (0.74 to 1.19).

Linkage disequilibrium (LD) was also assessed individually for the autosomal and the Z-chromosome datasets for the entire fall armyworm population, the C-strain, and the R-strain. No differences in patterns of LD were apparent for different chromosomes within the autosomes. Across all datasets, LD was higher on the Z-chromosome compared to the autosomes. As expected, all datasets exhibit a decay in LD as the distance between markers increases, with the asymptote for this decay occurring at a distance of 7,500 to 10,000 bp (Figure S2). However, when the entire fall armyworm population (C-strain and R-strain) is assessed together, LD remains elevated, especially on the Z-chromosome where median LD values were 0.195, more than 10-fold higher than any other dataset (Fig. 4C). Considered together, high values of nucleotide diversity, Tajima’s D, and LD on the Z-chromosome when both strains are present in the data, are a strong indication that two unique haplotypes (C-strain and R-strain) are being maintained on the Z-chromosome.

### Selective sweeps

**Fig. 5:**
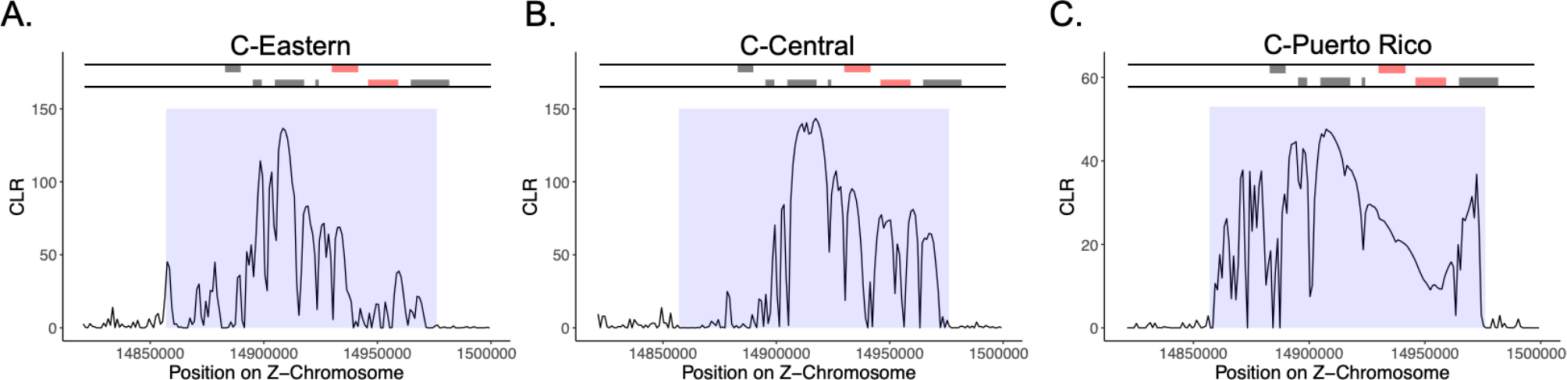
Composite likelihood ratios (CLR) calculated in 1 kb intervals within a Z-chromosome region that showed evidence of a selective sweep for all three C-strain sub-populations; **(A)** Eastern US, **(B)** Central US and **(C)** Puerto Rico. Lines above each graph represent specific coding sequence for genes in the genomic region located on the forward (top) or reverse (bottom) DNA strand. Genes in black are coding regions with unknown function. Genes in red indicate *Clk* (forward strand) and *Mdr49* (reverse strand)

Because geographic sub-structuring was present within the C-strain, we characterized selective sweeps within each distinct geographic C-strain sub-population (Central US, Eastern US, and Puerto Rico). Although they largely cluster with the C-strain central flyway, it is still unknown where individuals from the C-strain western flyway overwinter, and thus they were not included in this analysis. Using the SFS method, we found that a single region on the Z-chromosome showed evidence of selection in all three C-strain populations (Fig. 5). This region contained seven predicted coding regions, including two genes that had previously been annotated in an insect. The first was the gene encoding the circadian locomotor output cycles kaput protein (*Clk).* The second was the multidrug resistance protein homolog 49 (*Mdr49*). We did not find any evidence of selection within this region in the R-strain. Within the C-strain from the eastern US, we found two additional regions on the Z-chromosome with elevated CLR values, one of which corresponded to a glucose transporter (*Glut1*). The other contained two coding regions, neither of which have been functionally annotated in an insect species.

**Fig. 6:**
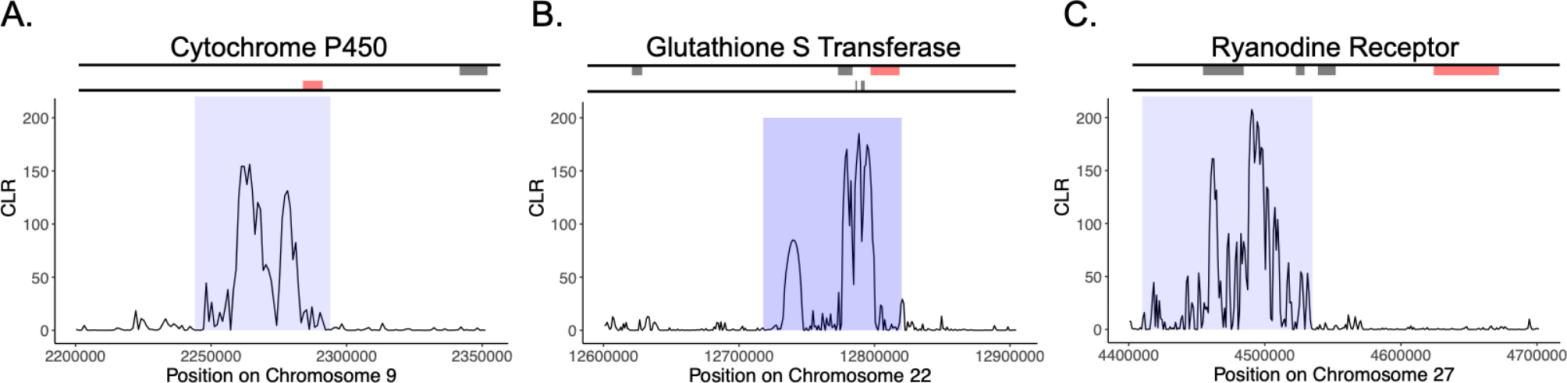
Genomic regions undergoing selective sweep in the C-strain eastern US sub-population. These regions were found on Chromosome 9 **(A)** Chromosome 22 **(B)**, and Chromosome 27 **(C)**. Lines above each graph represents annotated genes present within that genomic region that were located on the forward (top) or reverse (bottom) DNA strand. The genes colored in red indicate genes that could play a role in insecticide susceptibility and are indicated above the graph

On the autosomes, we found evidence that different geographic subpopulations and strains were experiencing unique selective pressures across genomic regions. The C-strain from the eastern US showed evidence of genetic hitchhiking on chromosomes 9, 22, and 27, indicating that these genomic regions may be undergoing a selective sweep (Figure S3). Notably, each of these regions corresponded with either a known gene involved in detoxification or a known insecticide target. The detoxification genes cytochrome p450 9e2 (*CYP9E2*) and glutathione S-transferase D7 (*GstD7*) were located within the outlier region on chromosome 9 and 22 respectively (Fig. 6A & B). Additionally, the gene encoding the Ryanodine Receptor gene (*RyR*) was downstream of the region under selection on chromosome 27 (Fig. 6C). It should be noted that each of these regions under selection also coded for an additional one to four genes. All genes that were present within the selected region are listed in Table S3.

Like the eastern US subpopulation of the C-strain, the central US C-strain subpopulation also showed evidence of increased selection on the region of chromosome 9 containing *CYP9E2*. Additionally, both C-strain subpopulations from the central US and Puerto Rico showed evidence of selection within the same region on chromosomes 30. This region contained four genes, two of which were previously annotated in *Drosophila melanogaster*: (1) Organic cation transporter-like protein (*Orct2*) and (2) the polycomb protein Scm (*Scm*). In addition to this region, chromosomes 10 and 25 were elevated in the central US subpopulation and chromosomes 15 and 23 were elevated in Puerto Rico. Within the R-strain we found additional outliers occurring on chromosomes 13, 14 and 18. Most genes annotated within these genomic regions are either proteins of unknown function or proteins that were identified in very distantly related species; therefore, their functional significance is unknown. All genomic annotations are presented in Supplementary Table S3.

The LD-method for selective sweep analysis identified one or two extreme outliers per population. In the eastern flyway subpopulation of the C-strain, this occurred in the same region on chromosome 27 near the Ryanodine Receptor gene (*RyR*) that was identified by the SFS method. Additional regions identified to be under selection for each population using the LD method can be viewed in Figure S4. Genes within these regions are also listed in Supplementary Table S3.

## Discussion

Understanding the population dynamics of insect pests, such as the fall armyworm, is informative for pest monitoring and management programs because it allows us to evaluate the risk of pest damage across regions and landscapes, track the potential spread of pesticide resistance, and develop models that better predict future pest infestations [59]. By analyzing polymorphisms derived from WGS data, we have shown that the primary genetic structure within the fall armyworm as a species in the US differentiates two previously described strains (C- and R-). Although this is consistent with previous findings [9, 11, 12], this strong pattern of differentiation has not always been observed with SNP data [57, 60]. Three non-mutually exclusive factors could explain this discrepancy; (1) misclassification of host strains, (2) underrepresentation of Z- linked SNPs, or (3) underrepresentation of the R-strain in previous datasets. For example, some studies classified the host strain for samples collected across both the Eastern and Western Hemispheres using only mtDNA markers. More recently, mtDNA markers were shown to be less reliable indicators of strain than Z-linked markers in the Western Hemisphere [12] and unreliable indicators of strain in the Eastern Hemisphere [61]. Misclassifications of strain based exclusively on mtDNA could have prevented researchers from identifying structure associated with strain. In our study, we only used samples collected in the Western Hemisphere, where strain-marker associations have been well established, and we used more reliable Z-chromosome markers to identify strain.

Although we found that most of the genetic variation in the dataset was explained by strain across both autosomal and Z-linked loci, this pattern was much stronger on the Z-chromosome. F_st_ values for loci on the Z-chromosome ranged from 0.6 to 0.7 between different strains across geographic regions. In contrast, F_st_ values across all autosomal markers ranged from 0.07 to 0.12 for the same comparisons. When we compared the entire fall armyworm population (C-strain + R-strain) with each individual strain separately, we found that having both strains in the dataset drastically increased the nucleotide diversity, Tajima’s D, and LD on the Z-chromosome. This is a strong indication that the Z-chromosome has a high level of linkage with a large group of linked SNPs routinely being inherited together and remaining within the C-strain or R-strain populations. As such, the Z-chromosome plays a disproportionate role in the genetic differentiation between strains. This may also explain why most of the previously described strain-specific makers have been located on this chromosome [42, 43]. Although F_st_ values were elevated across the entire Z-chromosome, absolute sequence divergence (Dxy) was predominantly elevated across one large region, or genomic island. This genomic island spans nearly the first half (10,000 kb) of the Z-chromosome. Dxy is unique from F_st_ in that it disregards within population diversity, and primarily reflects ancestral diversity [62]. Thus, it is likely that this region of the genome may contain genes involved in the initial divergence between strains. However, due to the length of this window and the large number of both putative and uncharacterized genes it contains, we cannot confidently identify a specific gene target that may have initiated strain divergence.

It has been suggested that the rate of structural rearrangements in lepidopteran genomes is one of the highest across all eukaryotes [63], and in a similar lepidopteran system, the European Corn Borer (*Ostrinia nubilalis*), the divergence of two strains (Z and E) was associated with an inversion in the Z-chromosome [63]. However, this does not seem to be the cause for genetic divergence between the fall armyworm strains. Despite strong differences between the Z-chromosome of the C-strain and the R-strain, we did not find evidence for a large inversion on this chromosome that could prevent recombination. As such, the fall armyworm may be more similar to the *Helicoverpa zea/ H. armigera* sister species system that exhibits substantial genomic differences on the Z-chromosome but lacks any detected chromosomal inversions [64].

Historically, researchers have tried to classify fall armyworm strains using differential phenotypes such as host plant use [9, 65] or allochronic behavior [12, 66]. However, differences in phenotype between strains are frustratingly inconsistent both within and between regions [67, 68]. As such, our data suggest that it is more accurate to consistently define these strains genetically as Z-chromosome strains rather than exclusively rely on phenotypic characterizations. Referring to these genetically distinct populations as Z-chromosome strains increases the identification accuracy without discounting the associated phenotypic differences or the need to infer their potential causal roles in strain divergence. This definition also explicitly implies that the best methods to identify strain involve using Z-linked markers such as the previously described *Tpi* [42] and Z-linked SNPs [43]. While slight differences in phenotype are still notable and may have important implications for pest management and evolutionary trajectories, the phenotypic overlap between strains has hindered our classification and understanding of these structured populations and how they have diverged. It is also likely that the differential phenotypes, including differences in host plant distribution and allochronic mating times, could be due to genes located on the Z-chromosome. This is supported by our selective sweep analysis that indicated all C-strain populations regardless of geography are experiencing selection on a region of the Z-chromosome that contains the circadian regulator gene, *Clk. Clk* plays a key role in maintaining the 24 h daily rhythm of many organisms, including in lepidoptera [69]. This gene likely plays an important role in circadian timing, including the timing of mating that has been implicated as a mechanism, maintaining reproductive isolation between the strains [10, 12, 66]. This gene was not present within the genomic island of strain divergence that we detected, indicating that if the *Clk* gene is responsible for allochronic mating times, selection for this differential phenotype may have arisen after the initial strain divergence.

Our data also suggest that the Z-linked strains (C- and R-) differ in their geographic ranges and population structure across the continent. Despite sequencing over 100 moths collected across multiple locations and time points in Arizona and California, we did not find evidence of R-strain individuals occurring in the western US. This is consistent with one previous observation [32] and again indicates that strains may exhibit differences in their dispersal and distribution patterns, with R-strain fall armyworms rarely if ever crossing the Rocky Mountain Range. This was especially surprising given that most of our collections within this western region occurred near pasture or alfalfa, which are host plants commonly associated with the R-strain. Although more years of sampling are needed to confirm this pattern, this would only be the second region identified in the Western Hemisphere where a single strain is found in isolation. To our knowledge, the only other region where strains have been surveyed and the R-strain has been absent is in Ecuador, in which most collections were done west of the Andes Mountain range [70]. More data are needed to better understand the factors that may be limiting R-strain individuals from occurring in these regions. Although moths collected across the western flyway were genetically similar to the C-strain moths from the central flyway, more studies with expanded sampling particularly in Mexico are also needed to better understand where fall armyworms in the west are overwintering, what dispersal patterns they are following, what they are feeding on, and their overall pest status within this region.

In addition to strain differences, our study also revealed unique sub-structuring between C-strain fall armyworm populations collected in different geographic ranges (Puerto Rico, the eastern flyway, and central/western flyways). This is the first indication that the overwintering populations in the US show genetic differentiation in the nuclear genome. This suggests that gene flow between the eastern and central flyways is insufficient to homogenize these populations completely. However, we found no evidence of geographic sub-structuring within the R-strain. This indicates that the R-strain exhibits higher levels of admixture across a broader geographic range than does the C-strain, which may have more distinct geographic populations. These results support some of the original suspicions of Pashley et al. (1987) [71] about the possibility of strain-specific population dynamics and movement patterns in the fall armyworm. This also suggests that the fall armyworm as a species is indeed comprised of two unique pests that should be monitored individually.

Additionally, these unique patterns between strains may explain why geographic sub-structing has not always been identified in previous studies of fall armyworm population structure in the Western Hemisphere [57, 72, 73]. Most of these previous studies relied on lower-resolution genetic markers, such as AFLPs, and grouped all fall armyworms, regardless of strain, into geographic regions. These studies found equal or higher genetic variation within a geographic region than between geographic regions and concluded that admixture was too high amongst fall armyworms across the entire Western Hemisphere to observe geographic structuring. However, this conclusion assumes that fall armyworm strains are either genetically similar or exhibit the same patterns of geographic sub-structuring. Our data suggest that neither of these assumptions is valid, and therefore, both strain and geography must be considered independently.

The only previous studies in the Western Hemisphere that have considered geographic patterns of structuring within a strain showed that the ratio of two common mitochondrial haplotypes within C-strain individuals could reliably differentiate between the eastern US flyway and the central US flyway [28]. These markers have since been used to identify the migratory trajectory of C-strain fall armyworms across the continent [27, 30, 31, 74]. However, no markers have been identified within the R-strain that exhibit similar geographic patterns. This is consistent with our findings that genetic isolation occurs between the central and eastern flyways within the C-strain, whilst the R-strain is more admixed across geographic ranges.

Our study also found that C-strain moths collected in Puerto Rico were a distinct population from those collected in the eastern flyway (F_st_=0.047). Using the previously described ratio of mitochondrial haplotypes, these two populations were considered the same due to similar ratios of common haplotypes [75]. Because these ratios are very low-resolution markers, it is likely they do not identify all patterns of geographic sub-structuring within the C-strain. Rather, haplotype ratios in Puerto Rico and the eastern flyway could be similar by chance, or because these populations derived from a similar historical population and have since diverged. Although we know the R-strain does occur in Puerto Rico [9], we did not have any R-strain represented in our collections and thus do not know the extent to which they are distinct from R-strain fall armyworms collected within the continental US.

Across the C-strain we found evidence that regions of the genome important for detoxification of insecticides may be experiencing selective sweeps. We also found that selection on these regions differed between geographic overwintering subpopulations. In both the central and eastern US C-strain subpopulations, we found evidence of selection acting on a CYP9 gene on chromosome 9. Cytochrome p450 monooxygenases family 9 (CYP9) genes are known to play a role in xenobiotic detoxification and have been implicated in insecticide resistance [76]. Additionally, the other two regions of the genome that showed patterns consistent with positive selection within the eastern US C-strain subpopulation were also located in and around genes that play roles in detoxification or insecticide modes of action. On chromosome 22, the glutathione S transferase gene (*GSTD7*) was closely linked to the genomic region under selection. GSTD7 plays a role in insecticide metabolism, including in resistance to imidacloprid in the whitefly, *Bemisia tabaci* [77]. Finally, the region under selection on chromosome 27 was located just upstream of the gene for the ryanodine receptor (*RYR*). RYR is the target site for diamide insecticides and mutations in this gene confer resistance to diamides in fall armyworm [38]. It should be noted that all genomic regions under selection contained more than one gene, so although it is an interesting trend that all regions under selection in the eastern US C-strain sub-population contain a gene implicated in insecticide resistance, we cannot definitively conclude this is due to selection for insecticide resistance. Still, the genomic patterns we observe indicate that the eastern flyway which is colonized by fall armyworm overwintering in south Florida (primarily Florida, Georgia, South Carolina, and North Carolina) may harbor populations with reduced insecticide susceptibility. Further studies should continue to monitor resistance to chemical insecticides across the C-strain subpopulations, with a special emphasis on differences between the two overwintering range that serve as source populations for the rest of North America.

## Conclusions

By implementing novel whole genome sequencing technologies, we have begun to unravel patterns of population structure, linkage, and selection across the genome of a notorious agricultural pest, the fall armyworm. Our data strongly support the identity of two unique genetically-distinct strains of fall armyworm in the US, commonly known as the C-strain and the R-strain. Although these strains are genetically distinct, differentiation is not uniform across the genome. The Z-chromosome is largely unique between strains and seems to comprise distinct C-strain and R-strain haplotypes. Thus, our data indicates these strains are most appropriately defined genetically as Z-chromosome strains. We found evidence that all C-strain populations regardless of geographic location were under selection in a region of the Z chromosome containing the circadian regulator gene *Clk.* This could underly differences in allochronic phenotypes that have been demonstrated between the strains. Our data also suggest that the strains exhibit unique geographic distributions and distinct patterns of geographic sub-structuring. These patterns strongly indicate that the two strains have different dispersal patterns and should be considered unique pests. Finally, our data reveal that both the central US and eastern US C-strain subpopulations may be under selection for a CYP detoxification gene that could alter the insect’s susceptibility to chemical insecticides. Two additional regions of the genome that may play a role in insecticide susceptibility also showed evidence of selection in the eastern US C-strain subpopulation. This could indicate that across the continental US, the C-strain is at greater risk of developing resistance to chemical control methods. This concern may be acutely elevated in the eastern flyway that is colonized by migrants that overwinter in south Florida. These strain-specific patterns of population structure should be considered when devising and implementing management strategies for this pest dyad.

## Electronic supplementary material

Below is the link to the electronic supplementary material.

Supplementary Material 1

## Data Availability

All sequence data and sample metadata that were generated and analyzed during the current study are available in the NCBI sequence read archive (SRA). These sequences can be retrieved using the BioProject ID PRJNA1115088, or at the following link: https://www.ncbi.nlm.nih.gov/bioproject/PRJNA1115088/.

## Abbreviations

WGS: Whole genome sequencing
US: United States
CYP: Cytochrome p450 monooxygenases
GST: Glutathione S-transferase
RYR: Ryanodine receptor
Bt: Bacillus thuringiensis
SNP: Single nucleotide polymorphism
PCA: Principal component analysis
LD: Linkage disequilibrium
CLR: Composite likelihood ratio
SFS: Site Frequency Spectrum
