# Supplementary material for: Genomic patterns of strain-specific genetic structure, linkage, and selection across fall armyworm populations
Source: BMC Genomics. 2025 Feb 7;26:116. doi: 10.1186/s12864-025-11214-8 (PMC11803928; doi:10.1186/s12864-025-11214-8)
Supplement: Supplementary file 1 — Supplementary Material 1 [file 12864_2025_11214_MOESM1_ESM.docx]

**Figure S1.** F_st_ values calculated in 10kb sliding windows across every chromosome in the Gimenez et al (2020) genome. F_st_ peaks, or regions with Fst values nearing 1, indicate regions that are divergent between the C- and R- strains. Each 10kb window contained an average of 3.46 SNPs (SEM 0.067).

**Figure S2.** Average linkage disequilibrium (R^2^) between SNP pairs located at varying distance intervals within the autosomes (A) or the Z-chromosome (B). Data is either combined across all sampled individuals, only C-strain, or only R-strain. Because R^2^ was much lower in the autosomes than on the Z-chromosome, the scale of the y-axis between panels.

**Figure S3.** CLR calculated using SweeD in 5kb sliding windows across the genome for three C-strain geographic regions (A) eastern flyway, (B) central flyway, and (C) Puerto Rico and (D) the combined R-strain population. Points marked in red are outliers that may indicate genomic regions undergoing a selective sweep.

**Figure S4.** Omega values calculated in 5kb sliding windows across the genome for three C-strain geographic regions (A) eastern flyway, (B) central flyway, and (C) Puerto Rico and (D) the combined R-strain population. Points marked in red are outliers that may indicate genomic regions undergoing a selective sweep.

**Table S1.** **Pairwise F_st_ values comparing fall armyworm strains across different flyways using only autosomal loci.** Values highlighted in grey indicate comparisons between strains.

|  | **C-West** | **C-East** | **C-Central** | **C-Puerto Rico** | **R-East** | **R-Central** |
| --- | --- | --- | --- | --- | --- | --- |
| **C-West** | - |  |  |  |  |  |
| **C-East** | 0.011 | - |  |  |  |  |
| **C-Central** | 0.005 | 0.011 | - |  |  |  |
| **C-Puerto Rico** | 0.027 | 0.03 | 0.028 | - |  |  |
| **R-East** | 0.091 | 0.076 | 0.089 | 0.111 | - |  |
| **R-Central** | 0.095 | 0.08 | 0.094 | 0.116 | 0.005 | - |

**Table S2.** **Pairwise F_st_ values comparing fall armyworm strains across different flyways using loci on the Z-chromosome**. Values highlighted in grey indicate comparisons between strains.

|  | **C-West** | **C-East** | **C-Central** | **C-Puerto Rico** | **R-East** | **R-Central** |
| --- | --- | --- | --- | --- | --- | --- |
| **C-West** | - |  |  |  |  |  |
| **C-East** | 0.041 | - |  |  |  |  |
| **C-Central** | 0.005 | 0.04 | - |  |  |  |
| **C-Puerto Rico** | 0.052 | 0.047 | 0.051 | - |  |  |
| **R-East** | 0.641 | 0.624 | 0.633 | 0.656 | - |  |
| **R-Central** | 0.7 | 0.685 | 0.693 | 0.715 | 0.036 | - |

**Table S3.** Known genes within the selective sweep regions for each population. Only genes that had previously been annotated in an insect are presented. Genes marked with astrisks are located just outside of the selection region. Chromosomes marked with two asterisks indicate no genes were annotated in closely related species (i.e. insects) within this region.

| **Flyway** | **Chrom** | **Selection Location** | **Selective Sweep Detection Method** | **Gene Symbol** | **Gene** | **Species Annotation** | **Gene Location** |
| --- | --- | --- | --- | --- | --- | --- | --- |
| **CS-Eastern** | 9 | 2248276-2288144 | SFS | CYP9E2 | Cytochrome P450 9e2 | *Blattella germanica* | 2283883-2291148 |
|  | 16** |  | LD |  |  |  |  |
|  | 22 | 12734162-12799128 | SFS | Gs1 | Glutamine synthetase 1, mitochondrial | *Drosophila melanogaster* | 12789902-12792743 |
|  |  |  |  | GstD7 | Glutathione S-transferase D7 | *Anopheles gambiae* | 12797028-12818462 |
|  | 27 | 4418973-4528648 | SFS & LD | path | Proton-coupled amino acid transporter-like protein pathetic | *Drosophila melanogaster* | 4454580-4484468 |
|  |  |  |  | RyR* | Ryanodine receptor | *Drosophila melanogaster* | 4624024-4672037 |
|  | 31 | 2174906-2209901 | SFS | Glut1 | Glucose transporter type 1 | *Drosophila melanogaster* | 2200875-2217357 |
|  | 31** | 5299495-5409480 | SFS |  |  |  |  |
|  | 31 | 14878105-14968092 | SFS | Clk | Circadian locomoter output cycles protein kaput | *Drosophila melanogaster* | 14929935-14941582 |
|  |  |  |  | Mdr49 | Multidrug resistance protein homolog 49 | *Drosophila melanogaster* | 14945910-14959191 |
| **CS-Central** | 1 | 2763266-2858275 | LD | wat | Fatty acyl-CoA reductase wat | *Drosophila melanogaster* | 2768611-2776988 |
|  | 9 | 2268210-2278177 | SFS | CYP9E2* | Cytochrome P450 9e2 | *Blattella germanica* | 2283883-2291148 |
|  | 10 | 1267043-1277051 | SFS | Ca-alpha1D | Voltage-dependent calcium channel type D subunit alpha-1 | *Drosophila melanogaster* | 1275753-1312430 |
|  | 25** | 3918816-3923764 | SFS |  |  |  |  |
|  | 30 | 5950262-5975242 | SFS | Orct2 | Organic cation transporter-like protein | *Drosophila melanogaster* | 5969918-5979683 |
|  |  |  |  | Scm | Polycomb protein Scm | *Drosophila melanogaster* | 5950107-5956835 |
|  | 31 | 14878105-14968092 | SFS | Clk | Circadian locomoter output cycles protein kaput | *Drosophila melanogaster* | 14929935-14941582 |
|  |  |  |  | Mdr49 | Multidrug resistance protein homolog 49 | *Drosophila melanogaster* | 14945910-14959191 |
| **CS-Puerto Rico** | 6** | 14031115-14036115 | LD |  |  |  |  |
|  | 15 | 5116174-5625621 | SFS | Dhc64C | Dynein heavy chain, cytoplasmic | *Drosophila melanogaster* | 5114354-5137738 |
|  |  | 5590659-5625621 | SFS & LD | eIF3-S6 | Eukaryotic translation initiation factor 3 subunit E | *Bombyx mori* | 5612960-5617599 |
|  | 23 | 664487-6834585 | SFS | AAEL008004 | Elongation of very long chain fatty acids protein AAEL008004 | *Aedes aegypti* | 6682887-6701082 |
|  |  |  |  | PPO2 | Phenoloxidase subunit 2 | *Bombyx mori* | 6713106-6719836 |
|  |  |  |  | SDR-1 | Farnesol dehydrogenase | *Aedes aegypti* | 6828252-6832291 |
|  | 30 | 5960254-5975242 | SFS | Scm | Polycomb protein Scm | *Drosophila melanogaster* | 5950107-5956835 |
|  |  |  |  | Orct2 | Organic cation transporter-like protein | *Drosophila melanogaster* | 5969918-5979683 |
|  | 31 | 14878105-14968092 | SFS | Clk | Circadian locomoter output cycles protein kaput | *Drosophila melanogaster* | 14929935-14941582 |
|  |  |  |  | Mdr49 | Multidrug resistance protein homolog 49 | *Drosophila melanogaster* | 14945910-14959191 |
